# Open Fracture Dislocation of the Talus With Partial Talar Extrusion: A Case Report

**DOI:** 10.7759/cureus.40673

**Published:** 2023-06-20

**Authors:** Gheorghe Cujba, Nicolae Angan, Mihaela Dragusanu

**Affiliations:** 1 Orthopaedic Surgery, Elias Clinical Emergency Hospital, Bucharest, ROU; 2 Trauma and Orthopaedic Surgery, Letterkenny University Hospital, Letterkenny, IRL

**Keywords:** talus fixation, talus avascular necrosis, open talus fracture, talus fracture dislocation, talus extrusion

## Abstract

We report a case of open talar fracture-dislocation (Gustilo-Anderson type IIIA) associated with a posterior tibial artery injury. The limb was aligned and splinted in the emergency department. In the operating theater, the posterior tibial artery was ligated, the talar neck fracture was reduced, and it was fixed with two Kirschner wires (K-wires). After K-wire removal, the patient underwent rehabilitation to regain function and resumed activities of daily living (ADL). At nine months of follow-up, the patient has a good ankle range of motion (ROM) and a congruent ankle joint but has developed avascular necrosis (AVN) of the talus.

This case report highlights the high risk of talus AVN after open talar fracture dislocation. Preservation of the extruded talus and anatomical reduction can maintain ankle alignment, which is essential for arthrodesis in cases of AVN complications.

## Introduction

The talus is an important anatomical structure connecting complex associations that link the ankle, hindfoot, and transverse tarsal joints [1]. Injuries to the talus of any kind can significantly affect the motion and function of any of those joints. Talar fracture dislocations are rare injuries, accounting for 0.06% of all dislocations and 2% of all talar injuries, and are usually associated with malleolar fractures or a talar fracture itself [2-3]. These types of injuries are usually caused by high-energy injuries with disruption of almost all ligaments and capsular attachments of the talus [4]. It usually leads to degenerative changes in the adjacent joints, and frequently, avascular necrosis (AVN) is a predictable outcome [5]. Despite advancement in the medical field, talus fractures continue to be a challenge for the treating orthopaedic surgeon due to the high number of complications associated with their treatment: AVN, nonunion, malunion, osteoarthritis, and infection [6].

## Case presentation

**Figure 1:**
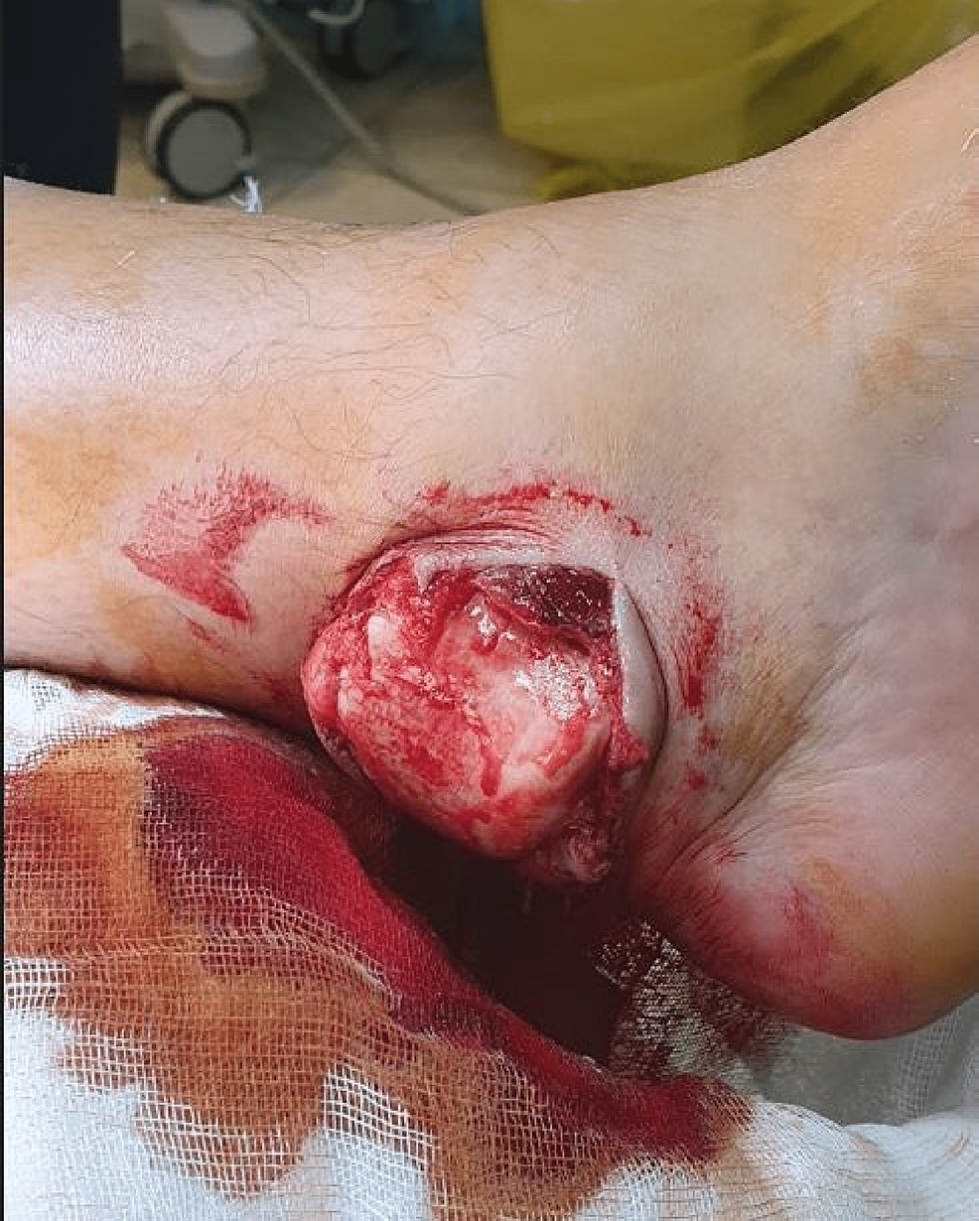
The talus dome is seen protruding through the wound in the posteromedial aspect of the left ankle

A 46-year-old man presented to the emergency department (ED) with complaints of left ankle pain and deformity after a building wall fell over his leg. The foot was entrapped, and he sustained a twisting injury that consisted of ankle plantarflexion and external rotation. The injury was isolated. Initial inspection revealed a bony protrusion through the wound on the medial side of the ankle (Figure 1).

**Figure 2:**
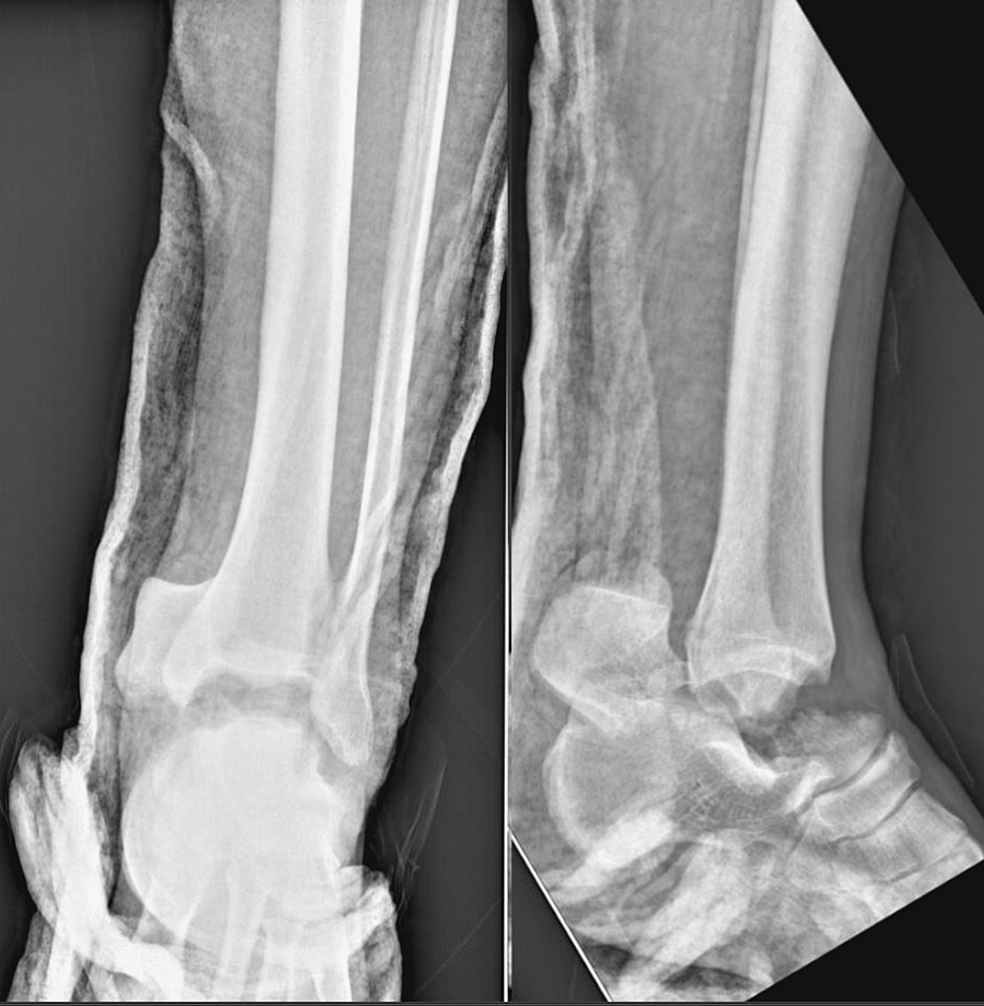
Anteroposterior (AP) and lateral X-rays of the left ankle

Physical examination revealed a palpable dorsalis pedis pulse, a wound at the posteromedial aspect of the ankle, and an intact sensation. Radiography showed a left talar neck fracture and enucleation of the body of the talus (Figure 2).

The patient received a tetanus vaccine, and broad-spectrum antibiotic therapy was initiated.

**Figure 3:**
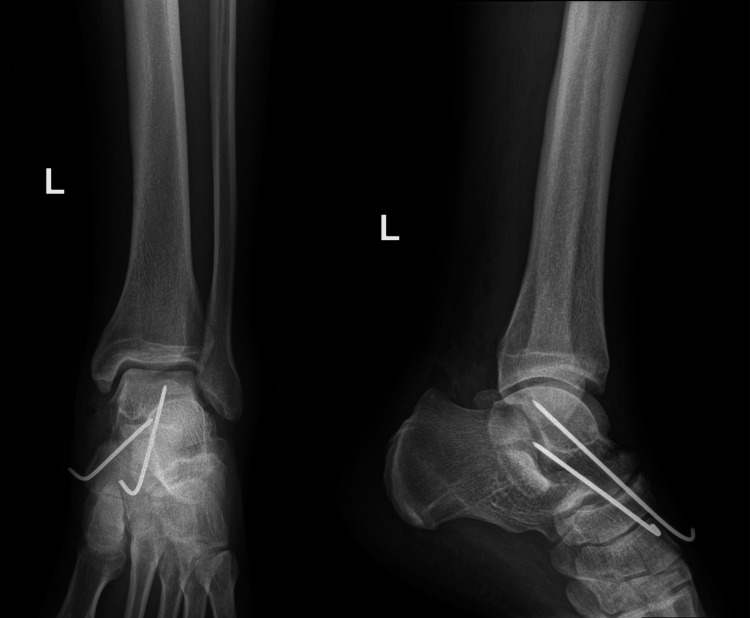
Anteroposterior (AP) and lateral view X-rays of the left ankle after surgery

The patient underwent a reduction and fracture-dislocation of the talus fracture, wound washout, debridement, and Kirschner wire (K-wire) placement under spinal anesthesia within six hours post-injury. The contamination was minimal. A Hook test was performed for possible associated syndesmotic injury, which showed stable syndesmosis. Intraoperatively, the X-rays were satisfactory. A complete tear of the posterior tibial artery was identified and ligated. The wound was sutured. The following day, a check X-ray was performed (Figure 3).

**Figure 4:**
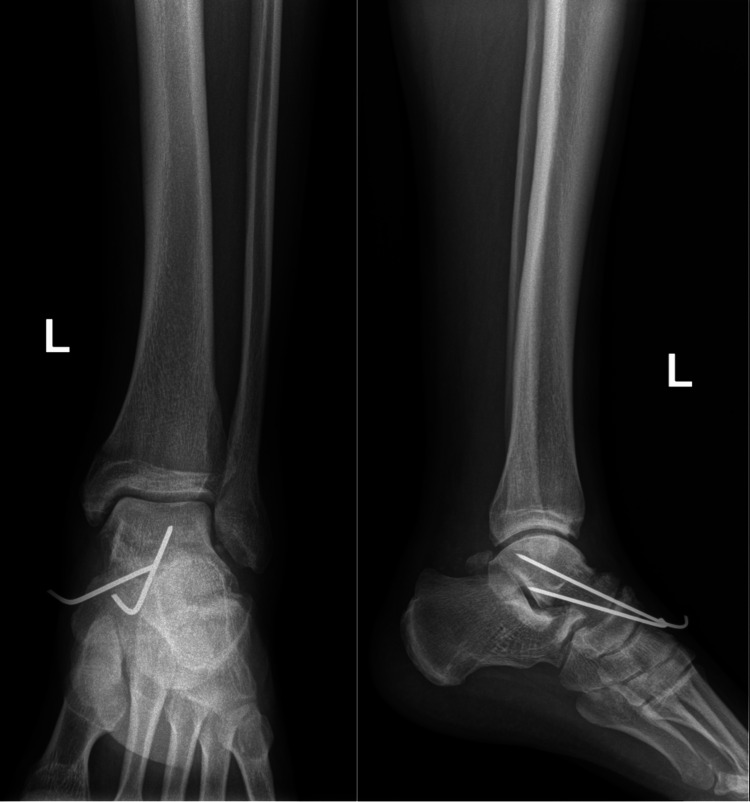
Anteroposterior (AP) and lateral view X-rays of the left ankle six weeks post-injury

Backslab immobilization was applied. The patient had subcutaneous enoxaparin (0.4 mL daily) for six weeks as an anticoagulant therapy. Two weeks after surgery, the backslab was changed to a non-weight-bearing (NWB) cast. The K-wires were removed at six weeks, and ankle range of motion exercises were initiated. A check X-ray was done six weeks post-injury (Figure 4).

Partial weight bearing was allowed at ten weeks and full weight bearing at three months post-injury.

**Figure 5:**
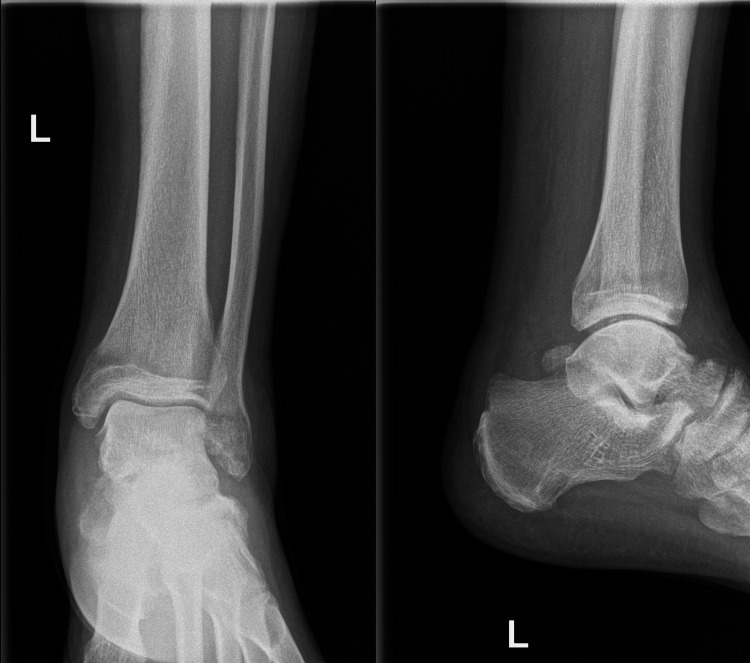
Anteroposterior(AP) and lateral view X-rays of the left ankle at three months

At three months follow-up, the patient started mobilizing full weight bearing and had minimal ankle pain. X-rays (Figure 5) show fracture healing and no signs of talar head AVN.

At six months follow-up, the patient had moderate ankle pain, especially after long periods of standing. X-rays didn’t show any ongoing changes. An MRI of the left ankle was done, which showed non-union of the fracture, multiple bone infarcts (osteonecrosis) at the level of the distal tibial metaphysis, and talocalcaneonavicular osteoarthritis (OA).

**Figure 6:**
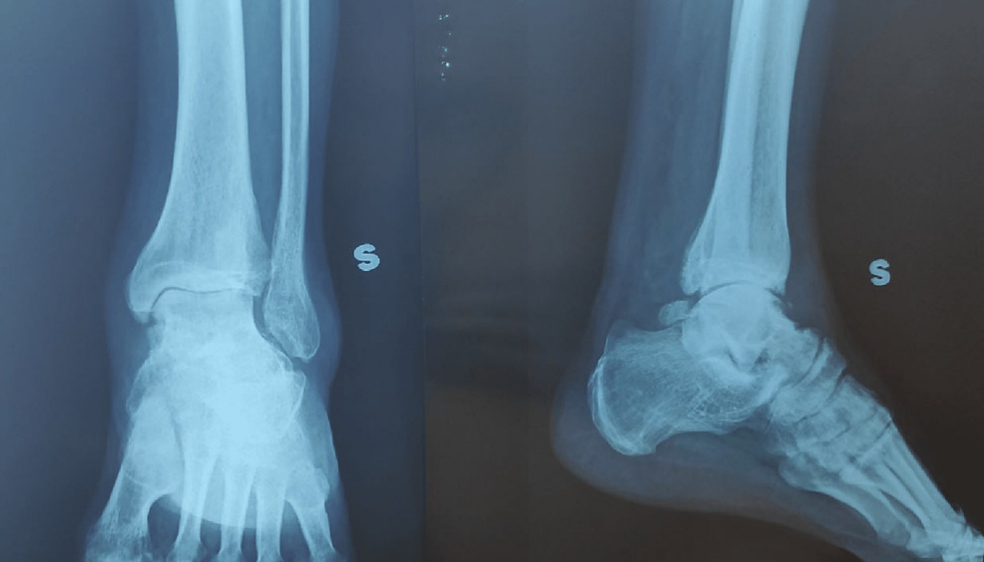
Anteroposterior (AP) and lateral view X-rays of the left ankle show ankle osteoarthritis, tarsal bones, osteoporosis, and talus avascular necrosis (AVN)

At nine months post-injury, the patient was complaining of mild ankle pain after long walks. The range of motion was ten degrees of dorsiflexion and thirty degrees of plantar flexion. An X-ray was performed, showing ankle, subtalar, and talonavicular osteoarthritis (OA) progression (Figure 6).

**Figure 7:**
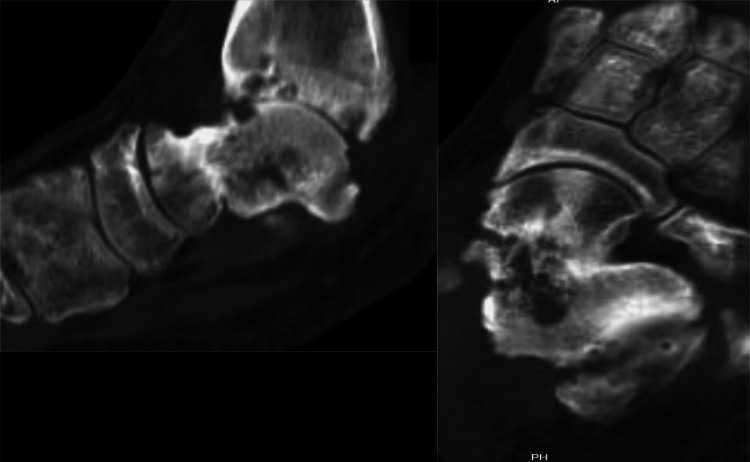
Sagittal and axial views of the CT scan of the left ankle

One year after surgery, a CT scan was done to assess the healing of the fracture (Figure 7). On axial and sagittal views, there is a callus. The tibiotalar joint space is obliterated, and subchondral cysts are present, which are suggestive of ankle osteoarthritis.

The patient received non-steroid anti-inflammatory drugs (NSAIDs), which improved the symptoms. We plan to perform triple arthrodesis if the patient develops significant pain due to OA progression in the future.

## Discussion

Extruded talus is a rare but serious injury with long-term implications [7-9]. The talus is usually not completely detached. If some of the ligaments are torn, the talus can be partially expelled from the wound, as in our case.

**Figure 8:**
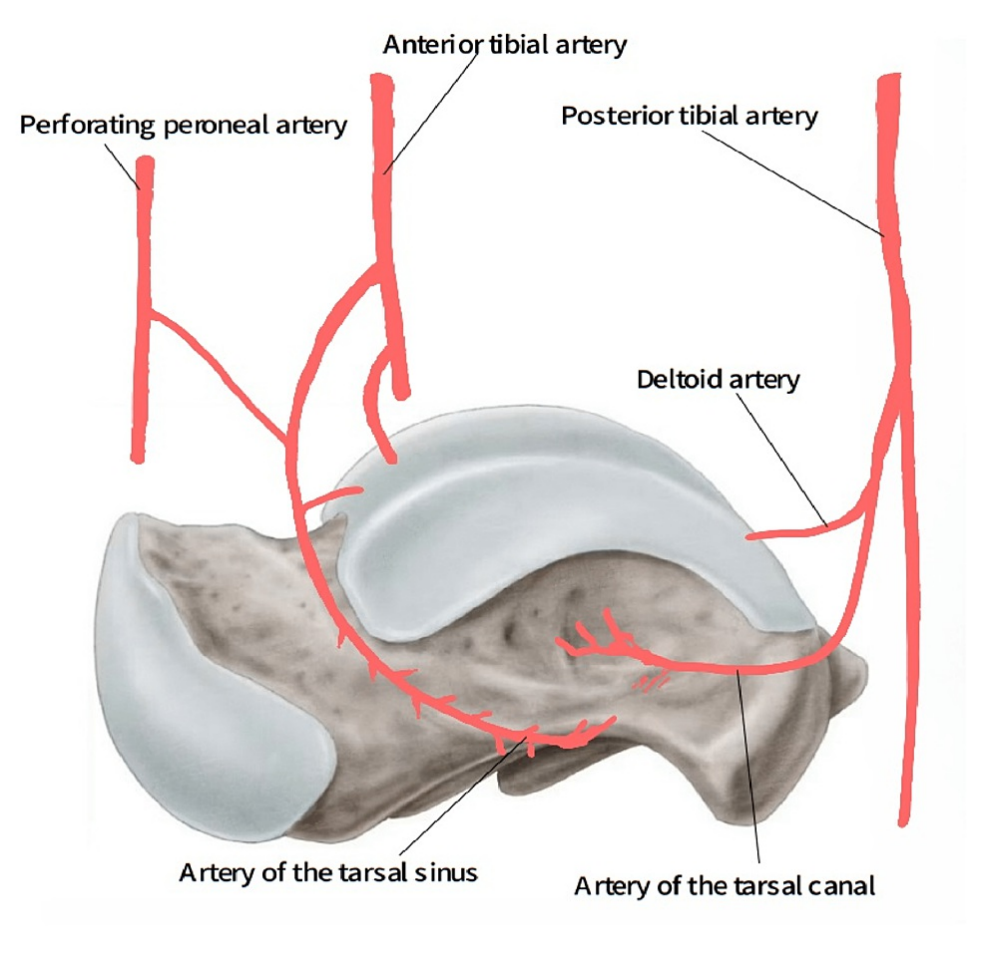
Blood supply to the talus

The blood supply to the talus is insubstantial. Approximately 60% of the talus is covered by articular cartilage, leaving only a small area for blood vessels to penetrate. This characteristic increases the vulnerability of the talus to avascular necrosis [10]. The artery of the tarsal canal, supplied by the posterior tibial artery, forms an anastomotic sling with the artery of the tarsal sinus (Figure 8).

This arrangement contributes to the blood supply of the inferior aspect of the neck and the posterior region of the talus body. Many experts consider this vascular source to be crucial, as it provides blood flow to approximately two-thirds of the lateral aspect of the talus body [11, 1]. Additionally, the posterior tibial artery gives rise to a deltoid branch that travels through the ligament and supplies blood to the medial body of the talus [11]. As the anterior tibial artery extends distally, it provides vascular branches that provide blood supply to the dorsal aspect of the neck and some parts of the dorsal aspect of the talar head [11]. These vessels are susceptible to injury when there is dorsal impaction of the neck against the plafond, like in talar neck fractures.

The first description of blood supply to the talus was made by Wildenauer and then by Haliburton in the 1950s [12]. The arteries that supply the talus are the anterior tibial, the posterior tibial, and the perforating peroneal arteries [13]. The contribution of the anterior tibial artery is estimated to be around 36% to the blood supply of the talus, while the posterior tibial artery accounts for approximately 47%, and the perforating peroneal arteries make up roughly 16%.

Open fracture dislocation of the talus requires urgent irrigation, debridement, and fracture reduction [5,14,15]. Urgent reduction and careful sot-tissue handling minimize skin complications [1,16,17]. The worst possible complication of open fractures is infection, which can lead to prolonged treatment or amputation.

Stabilization is typically performed with K-wire pinning to stabilize the talus at both the subtalar and the talonavicular joints [18].

Assessing healing through regular X-rays can be challenging, so CT scans can be performed to assess the healing process. It's crucial to follow up with the patient 36 months after fixation for the possible AVN complication.

Although AVN is present, it has the potential to improve, and the talus can undergo remodeling even as late as 36 months after the injury. Prolonged non-weight-bearing, therefore, has not been proven to be beneficial [19]. Actually, even if AVN and sclerosis of the dome are present, the talus has been shown to be sufficiently strong to support weight despite the present obesity. Patients in this situation may not experience any problems until the talus revascularizes, which can take several months or years.

The possible long-term complication is talus dome collapse due to full weight bearing, which may clinically manifest as new ongoing pain. Triple fusion may be necessary in this case.

## Conclusions

The alignment of the foot and ankle is satisfactory, which is important for possible future fusion. The overall evolution of the case was good, although mild chronic pain was present.

Early reduction with irrigation and debridement should be performed in this type of injury, and great care should be taken to preserve the extruded talus because the results are significantly better with procedures that retain the talar body. Wound infection and osteomyelitis are serious conditions that can change therapeutic management and lead to limb amputation.
